# Agreement on Indication for Systemic Therapy between Biopsied Tissue and Surgical Excision Specimens in Breast Cancer Patients

**DOI:** 10.1371/journal.pone.0091439

**Published:** 2014-03-10

**Authors:** Alexander M. Th. Schmitz, Joost J. Oudejans, Kenneth G. A. Gilhuijs

**Affiliations:** 1 Department of Radiology, Image Sciences Institute, University Medical Center Utrecht, Utrecht, The Netherlands; 2 Department of Pathology, Diakonessen Hospital, Utrecht, The Netherlands; University General Hospital of Heraklion and Laboratory of Tumor Cell Biology, School of Medicine, University of Crete, Greece

## Abstract

**Purpose:**

In breast cancer therapy, the indication for systemic therapy is typically based on prognostic markers from the surgical excision specimen. If such is unavailable, for instance prior to tumor ablation therapy, the indication for adjuvant systemic therapy may be assessed from pretreatment biopsies. The effect of differences in tumor characteristics between biopsy and excision specimen on agreement in indication for systemic therapy is, however, largely unknown. The aim of this study is to determine the agreement in eligibility for systemic therapy between preoperative and postoperative assessment. Secondly, to identify which patient-, tumor- or lymph node characteristics may influence this agreement.

**Materials and Methods:**

In this retrospective study, 300 consecutive female patients with primary invasive breast carcinoma on biopsy were included. Indication for systemic therapy was determined separately from biopsied tissue and from excision specimens using national guidelines based on adjuvant! online. Agreement was assessed, and patient-, tumor- and lymph node characteristics affecting agreement analyzed.

**Results:**

Agreement in tumor characteristics between biopsy and excision specimen varied from high (ER-status: 99%), to lower (tumor grade: 62%, tumor size: 59%, and lymph node status: 67%). Without preoperative sentinel node biopsy (SNB), agreement on systemic therapy exists in 77% of patients (kappa = 0,547). Positive pretreatment indication for systemic therapy was highly indicative of the postoperative indication (PPV = 94%). Negative indication was, however, only indicative in 67% of patients. Conversely, with preoperative SNB, agreement on negative indication was raised to 89%. Agreement was especially high for ER-negative status, tumor grade 3, tumor size >2 cm, lymph node positivity at biopsy and a negative preoperative SNB.

**Conclusions:**

A positive indication for systemic therapy from biopsy is highly indicative for a positive indication from the excision specimen. When the indication is negative, additional stratification using preoperative SNB raises the agreement. Still, discordance occurs in 1 out of 10 patients.

## Introduction

Breast cancer therapy is a combination therapy aiming to balance local control with therapy side effects. Planning of the optimal treatment is based on prognostic patient- and tumor characteristics. Pretreatment imaging and biopsy are performed to confirm the diagnosis of breast cancer, followed by surgery, radiotherapy, and systemic therapy in selected patients based on prognostic markers in the excision specimen. For this purpose, a well-established model such as adjuvant! online (AOL) can be used.

In the past decades, developments have led to less invasive breast cancer therapy in appropriately selected patients while maintaining survival rates: from mastectomy to breast-conserving surgery, from axillary lymph node dissection (ALND) to sentinel node biopsy (SNB) [1]–[6]. Progression towards more individualized therapy, using minimally-invasive techniques that destroy the tumor *in-situ* such as high-intensity focused ultrasound, cryoablation, radio-frequency ablation and preoperative radiotherapy [7]–[13] has shown to be feasible. However, progress is impeded by uncertainty about patients’ eligibility for adjuvant therapy, caused by the absence of a representative surgical specimen. To avoid under- or overtreatment, it is essential that the clinical introduction of primary minimally-invasive therapies is accompanied with accurate means to establish indication for systemic therapy in the absence of an excision specimen.

Disagreement between tumor characteristics derived from preoperative large core needle biopsy (LCNB) and excision specimen is often seen [14]–[20]. For tumor grade, an important prognostic marker, disagreement between LCNB and the excision specimen has been observed in around 40% of the patients [14], [15]. Other preoperative tumor characteristics, such as tumor size from imaging, show disagreement with the excision specimen in 30–50% of patients, dependent on the method of detection [21]–[23]. Preoperative assessment of lymph node status, based on axillary ultrasonography with guided fine-needle aspiration (FNA), has high specificity, but relatively low sensitivity [24], [25]. Therefore, indication for systemic therapy is typically still based on excision specimens.

With disagreements in tumor characteristics being widely recognized, to the best of our knowledge, it is yet unknown which impact these disagreements have on assessment of indication for systemic therapy. Possibly, patient subgroups exist where pretreatment assessment of indication for systemic therapy is more accurate than in other subgroups. Such knowledge would greatly contribute to further dissemination of individualized cancer treatment.

The aim of this study is to establish the agreement in patient eligibility for systemic therapy between preoperative assessment based on biopsy and postoperative assessment based on the excision specimen. Secondly, to identify which patient-, tumor- or lymph node characteristics may influence this agreement.

## Materials and Methods

### Patients

Ethics statement: In the Netherlands, retrospective scientific medical research on medical records analyzed anonymously is waived from formal medical ethics review and informed consent is not needed. This is set forward by the Medical Research Involving Human Subjects Act (WMO), confirmed by the central committee on research involving human subjects (CCMO), and can be read at www.ccmo-online.nl.

In this retrospective study, consecutive female patients with histologically proven invasive adenocarcinoma of the breast, who underwent ablation, amputation or lumpectomy (Table 1), were included between May 2009 and January 2013 in the Diakonessen Hospital in Utrecht. Exclusion criteria were residual, bilateral or multicentric disease, patients with T4-features, or patients treated with neo-adjuvant chemotherapy.

**Table 1:** Baseline characteristics.

Characteristics	N	%
Patients		
Total	300	100%
Age (years)		
≤54	91	30%
55–69	111	37%
≥70	98	33%
Origin of tumor size biopsy-stage		
MRI	49	16%
Ultrasound	231	77%
Mammography	12	4%
Physical Examination	8	3%
Tumor type excision-stage		
IDC	237	79%
ILC	47	16%
Other	16	5%
Type of surgery		
Ablatio	86	29%
Amputation	22	7%
Lumpectomy	192	64%
Axillary procedure biopsy-stage (positive)		
Fine-needle aspiration	49 (28)	
Biopsy	4 (4)	
Axillary procedure excision-stage (positive)^1^		
Sentinel node biopsy	258 (99)	
Axillary lymph node dissection	80 (50)	
None	9	
Nodal status excision-stage		
N0	168	56%
≥N1	132	44%

^1^More than 300 cases due to overlap between sentinel node biopsy and axillary lymph node dissection.

Patient-, tumor- and treatment characteristics of all 300 consecutive female patients with histologically proven invasive adenocarcinoma of the breast who were included in this study.

### Tissue and Pathology

Ultrasound-guided LCNBs were taken from the tumor during routine diagnostic workup. The preoperative lymph node status was assessed using axillary ultrasonography. FNA or biopsy was performed in radiological suspicious lesions [26]. Harvested tissue was fixed in neutral buffered formaldehyde and processed to paraffin blocks. Microscopic sections were taken and stained using hematoxylin and eosin (H&E).

Surgery was combined with SNB if no lymph node metastases were found preoperatively. If lymph node metastases were found preoperatively surgery was combined with ALND. Up until 2010 a positive SNB at surgery was followed by ALND, after 2010 this was only performed on indication (Table 1).

At pathology, the largest diameter of the tumor was assessed macro- and microscopically after sectioning the excision specimen in 4 mm thick slabs. Microscopic sections of the invasive tumor and lymph nodes were taken and stained using H&E.

### Patient and Tumor Characteristics

Tumor characteristics were collected from preoperatively available information (denoted “biopsy-stage”) and from postoperative information (denoted “excision-stage”). This information included estrogen receptor (ER)-status, tumor size, tumor grade, and lymph node status.

Age was derived from the date of surgery. ER-status was taken from the pathology records. A threshold of 10% staining was used to discriminate between ER-negative status (<10% staining) and ER-positive status (≥10% staining). Tumor grade from core biopsy was revised by a breast pathologist (JO). For the excision-stage, tumor grade was derived from pathology records. Carcinomas were graded 1, 2, or 3, according to the modified Bloom and Richardson guidelines [27]. These guidelines consider morphologic characteristics of tubule and gland formation, nuclear pleomorphism and mitotic count. The latter was based on 7 high-power fields (HPFs), corresponding to a field of view of 2 mm^2^. When the amount of core tissue was less than 7 HPFs, the mitotic count was extrapolated to 7 HPFs. Tumor size at the biopsy-stage was derived from the best available imaging examination. Largest tumor diameter measured at MRI was considered first, followed by that from ultrasound and mammography. If tumor size was not available from imaging, the size estimated from physical examination was used. At the excision-stage, the largest tumor diameter was taken from the excision specimen. Lymph node positivity was preoperatively examined using axillary ultrasound with or without FNA or biopsy. At the excision-stage, the best available evidence was used with preference for the results from ALND. Sentinel lymph nodes were defined positive if any isolated, micro- or macrometastases were visible on H&E-stained slides. The SNB status was unknown prior to surgery, but a preoperative SNB status was simulated by combining lymph node status from the biopsy-stage with SNB status from the excision-stage.

### Indication for Systemic Therapy

For every patient, indication for systemic therapy was assessed separately at the biopsy-stage and at the excision-stage, based on AOL.

The web-based tool AOL (version 8.0) estimates risk-percentages of mortality and relapse based on the SEER tumor registry database, and has been validated on Canadian and Dutch populations [28]–[30]. The risk of mortality and relapse was based on the characteristics: age, ER-status, tumor grade, tumor size, and number of positive lymph nodes. Co-morbidity was set to default ‘minor problems’. At the biopsy- and excision-stage, the 10-year risk of breast cancer specific mortality and relapse was established. The indication for systemic therapy was based on the Dutch national guidelines, advising adjuvant systemic therapy when it reduces the 10-year risk of breast cancer specific mortality by at least 5%. In clinical practice, these guidelines roughly translate to risk of 10-year-mortality equal to or exceeding 15% or risk of 10-year relapse equal to or exceeding 25% (www.oncoline.nl).

In both stages, patients were divided into subgroups with and without indication for systemic therapy. The indication for systemic therapy at the excision-stage was considered to be the clinical standard.

### Analyses

Analyses were performed using SPSS (version 19.0). ER-status, tumor grade, tumor size and lymph node status known at the biopsy-stage were compared with those known at the excision-stage. Overall agreement between these characteristics was expressed as a percentage on the Kappa test. Agreement on indication for systemic therapy between the stages was established using Cohen’s kappa. In addition, the positive predictive value was calculated (PPV: the fraction of tumors that is correctly assumed to be indicated for systemic therapy at the biopsy stage), as well as the negative predictive value (NPV: the fraction of tumors that is correctly assumed not to be indicated for systemic therapy at the biopsy stage). Subgroups were stratified, and McNemar tests were used to assess agreement. In addition, a multivariate decision tree was constructed in order to help decide when the preoperatively known characteristics are likely to result in similar indication for systemic therapy as the postoperatively known characteristics. For this purpose, the dichotomous outcome variable “indication for systemic therapy” at the excision-stage was taken as the dependent variable. Independent variables were eligibility for systemic therapy at the biopsy-stage (forced first variable), age, ER-status, tumor grade, tumor size, and preoperative SNB status. The CHAID growing method was used. Backward covariate selection (p-to-remove = 0,05) was employed to determine under which conditions the indication for systemic therapy from core biopsy agrees with that from the excision specimen.

## Results

### Patients

**Figure 1:**
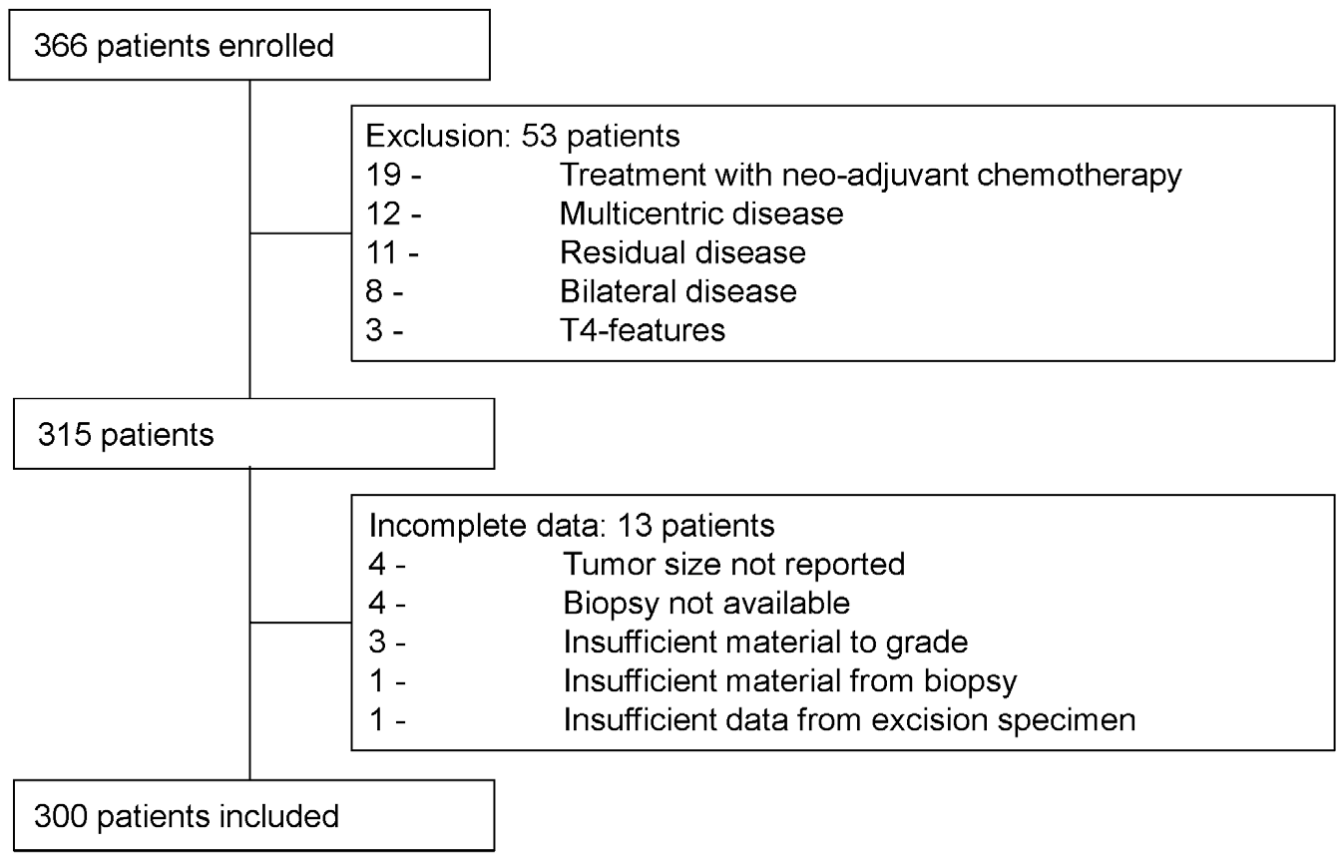
Patients enrolled, excluded, or removed from the study due to incomplete data.

Three hundred and sixty six patients were enrolled in our study; 53 patients were excluded and 13 patients had missing data (Figure 1). A total of 300 patients were finally included. The mean patient age at surgery was 62 years (range 34–89 years) (Table 1).

### Tumor Characteristics

The ER-status showed very high agreement between the biopsy-stage and the excision-stage (99%; K = 0,955) (Table 2). Tumor grade resulted in a lower agreement (62%; K = 0,345), with a comparable ratio of under- and overestimation (22%; N = 65 and 16%; N = 49, respectively). More agreement was seen in grade 1 and 2 tumors compared to grade 3 tumors (Table 3). Tumor size at biopsy-stage was most frequently derived from ultrasound examination followed by MRI, mammography and physical examination (Table 1). Agreement was found in 176 patients (59%; K = 0,406), although underestimation of size at the biopsy-stage occurred twice as often as overestimation (N = 84; 28%, and N = 40; 13%, respectively) (Table 4). Concerning lymph node status, a positive result at FNA or biopsy was always indicative of a positive result at the excision-stage (100%; N = 32). However, a negative result was less indicative (63%; N = 168), resulting in relatively poor overall agreement (67%; K = 0,264) (Table 5).

**Table 2:** Agreement between the biopsy-stage and excision-stage for estrogen receptor-status.

Biopsy-stage	Excision-stage	
Estrogen receptor-status	Positive	Negative
Positive	**259 (99%)**	1
Negative	2	**38 (97%)**
Total	261	39

**Table 3:** Agreement between the biopsy-stage and excision-stage for tumor grade.

Biopsy-stage	Excision-stage		
Tumor grade	Grade 1	Grade 2	Grade 3
Grade 1	**69 (62%)**	39	2
Grade 2	43	**101 (69%)**	24
Grade 3	0	6	**16 (38%)**
Total	112	146	42

**Table 4:** Agreement between the biopsy-stage and excision-stage for tumor size.

Biopsy-stage	Excision-stage				
Tumor size (cm)	0,1–1,0	1,1–2,0	2,1–3,0	3,1–5,0	>5,0
0,1–1,0	**37 (71%)**	35	3	0	0
1,1–2,0	13	**87 (63%)**	25	2	1
2,1–3,0	2	15	**35 (50%)**	17	1
3,1–5,0	0	2	6	**15 (43%)**	0
>5,0	0	0	1	1	**2 (50%)**
Total	52	139	70	35	4

**Table 5:** Agreement between the biopsy-stage and excision-stage for lymph node status on fine-needle aspiration or biopsy.

Biopsy-stage	Excision-stage	
Lymph node status	Positive	Negative
Positive	**32 (24%)**	0
Negative	100	**168 (100%)**
Total	132	168

### Indication for Systemic Therapy

Indication for systemic therapy was derived from AOL. Postoperatively, 55,7% of patients were indicated for systemic therapy. The indication derived from the biopsy-stage (without SNB) agreed with that from the excision-stage in 230 patients (77%; K = 0,547). A positive indication at the biopsy-stage nearly always corresponded with a positive indication at the excision-stage (PPV = 94%). Conversely, a negative indication was only negative at the excision-stage in approximately two thirds of the number of patients (NPV = 67%) (Table 6).

**Table 6:** Agreement between indication for systemic therapy at the biopsy-stage and excision-stage.

Adjuvant! online	Excision-stage		
Biopsy-stage	Systemic Therapy	No Systemic Therapy	Agreement: N (%); significance; kappa (95% CI)
Systemic Therapy	104	7	N = 230 (77%); p<0,001; K = 0,547 (0,461–0,633)
No Systemic Therapy	63	126	

Agreement in indication using adjuvant! online without preoperative sentinel node biopsy.

### Agreement in Subgroups

Table 7 stratifies the agreement on indication for systemic therapy in subgroups. Age did not affect the agreement, but ER-status did. ER-negative tumors led to more agreement than ER-positive tumors, despite the observation that ER-status was not significantly different between the biopsy-stage and excision-stage. Also, high-grade tumors led to stronger agreement, up to 100% in grade 3. Considering tumor size, the agreement was smallest in tumors ≤2,0 cm. Stronger agreement was found in patients with a positive lymph node status at the biopsy-stage. Conversely, a negative preoperative SNB status led to stronger agreement on indication for systemic therapy than a positive preoperative SNB.

**Table 7:** Agreement within subgroups of biopsy-stage tumor characteristics.

Tumor characteristics in biopsy-stage	Number of patients	Agreement with AOL^2^	
	N	N	%
Age (years)			
≤54	91	68	75%
55–69	111	81	73%
≥70	98	81	83%
ER-status			
Negative	39	35	90%^3^
Positive	261	195	75%
Tumor grade			
Grade 1	110	77	70%
Grade 2	168	131	78%
Grade 3	22	22	100%^3^
Tumor size (cm)			
0,1–1,0	75	55	73%
1,1–2,0	128	89	70%
2,1–3,0	70	61	87%^3^
3,1–5,0	23	21	91%^3^
>5,0	4	4	100%^4^
Lymph node status			
Negative	268	200	75%
Positive	32	30	94%^3^
Preoperative SNB^1^ status			
Negative	169	150	89%^3^
Positive	131	80	61%

1 Sentinel node biopsy;

2 Adjuvant! online;

3 Significant at McNemar test;

4 All patients were indicated for systemic therapy at the biopsy-stage.

Agreement in subgroups of tumor characteristics: age, estrogen receptor-status, tumor grade, tumor size, lymph node status on fine-needle aspiration or biopsy and preoperative sentinel node biopsy status.

### Multivariate Analyses

**Figure 2:**
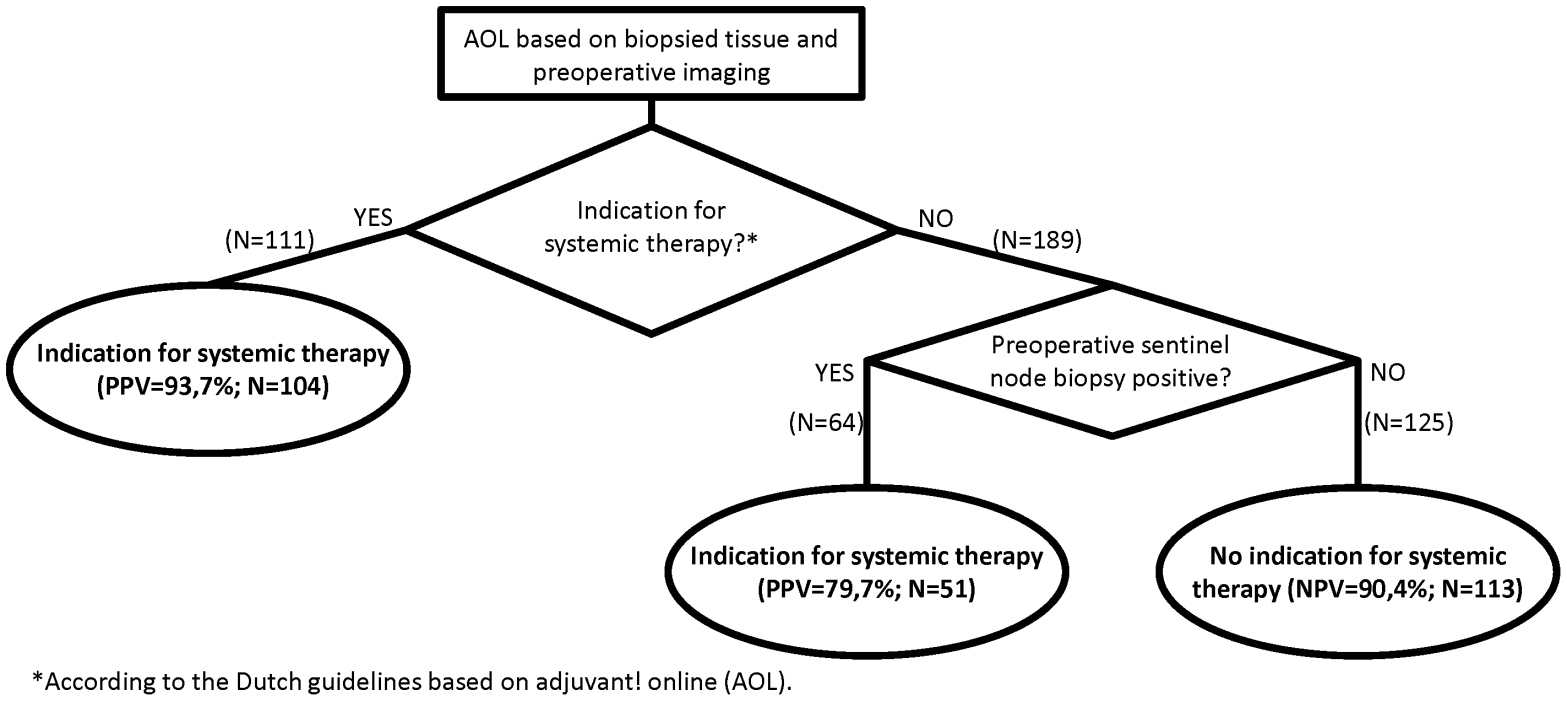
Decision tree based on AOL (N = 300) to establish confidence in preoperative indication for systemic therapy. Patients are divided into a positive- and negative indication group. For patients with a negative indication, a preoperative sentinel node biopsy raises the agreement.

Using multivariate analysis, a preoperative decision tree was created. Using biopsy-stage tumor characteristics in AOL, a subgroup containing 37% of patients was indicated for systemic therapy with high positive predictive value (PPV = 93,7%; N = 104). Combined with a preoperative SNB, 58,3% of the patients were indicated (PPV = 88,6%; N = 155). The remaining group of patients (41,7%) was selected without indication for systemic therapy (NPV = 90,4%, N = 113) (Figure 2).

## Discussion

In this study, agreement on indication for systemic therapy established from pretreatment biopsy and the surgical excision specimen was established and compared in subgroups of patients. Without preoperative SNB, agreement on systemic therapy exists in approximately 75% of the patients. A positive indication for systemic therapy, based on AOL guidelines using preoperative biopsies alone, was found to be highly indicative of a positive indication based on the surgical excision specimens. A negative preoperative indication was, however, only indicative in roughly two thirds of selected patients. With a preoperative SNB, this agreement was raised to approximately 90%.

Still, in approximately 1 of 10 patients disagreement in preoperative indication for systemic therapy exists. In the current study, using AOL, a subgroup of 64 patients with a positive preoperative SNB contained 13 patients who had no indication for systemic therapy postoperatively. The expected reduction in breast cancer mortality risk by systemic therapy ranged between 0,6% and 3,0% in these 13 patients.

The agreement on indication for systemic therapy was highest for characteristics associated with a poor tumor prognosis: ER-negative tumors, grade 3 tumors, tumors size >2cm and lymph node positivity at biopsy. Conversely, high agreement was also found for a negative preoperative SNB status, associated with a favorable prognosis in patients with a negative indication. Age was not of influence on agreement.

The observed disagreement on indication for systemic therapy results from differences in tumor characteristics between core biopsy and the surgical excision specimen, amplified or dampened by the biological process that underlies the prognostic model AOL. In AOL, the number of positive nodes, together with tumor size, are the major factors used to estimate patient prognosis [28].

ER-status derived from core biopsy agrees well with ER-status derived from the excision specimen (99%), which is also observed in the literature [19]. Agreement on tumor grade was, however, lower, comparable with the 60% agreement reported in the literature [14], [15], with lowest agreement on grade 3 tumors. This disagreement is probably caused by the heterogeneity of breast cancers, and the fact that LCNB samples only a small part of the tumor. The size and number of cores may be an influencing factor, but a previous study showed that the amount of tissue obtained from biopsy did not affect the agreement between core biopsy and excision specimen [14]. Agreement on tumor size of around 60% was seen, which is comparable with results from previous studies [21]–[23]. The lowest agreement on tumor size was seen in larger tumors (>2cm). As expected from prior studies [24], [25], a positive preoperative lymph node status derived from ultrasound-guided FNA or biopsy was highly correlated with a positive postoperative lymph node status, but a negative preoperative status was poorly associated.

Typically, the indication for systemic therapy is determined postoperatively from the excision specimens using AOL. We investigated whether postoperative tools such as AOL can be used to establish the indication for systemic therapy preoperatively. The decision tree illustrates the optimal strategy to accomplish this based on statistical considerations. The findings in this study will not impact the standard procedures for indication of systemic therapy but may have impact when experimental treatments such as high-intensity focused ultrasound, cryoablation, radio-frequency ablation or preoperative radiotherapy are used.

A limitation of our study is the number of patients. Although this number is relatively large compared to other reproducibility studies, results should be verified in studies with larger patient numbers. In addition, the decision tree devised in this study should undergo validation in an independent patient group. It should also be noted that AOL applies only after conventional breast cancer therapy, i.e., surgery, followed by radiotherapy. It is currently unknown whether minimally invasive techniques will lead to similar outcomes even if the patient selections are optimal. In the absence of any evidence on this point, it seems, however, a reasonable assumption to make at this time to advance further studies in this field, provided that these minimally invasive techniques are still followed up by radiotherapy.

In this study all characteristics were retrieved from the regular clinical setting, with the exception of tumor grade from LCNB, and are thus subject to inter-observer variation both at radiology and at pathology. Previous studies have already demonstrated the impact of inter-observer variation on patient selection for systemic therapy [31], [32]. Although these variations possibly also influenced the agreement in our study, the advantage of using data derived under clinical conditions is that it provides realistic estimations of the pretreatment performance of AOL in clinical setting.

For the indication of systemic therapy, AOL was used. This prognostic model is based on patient and tumor characteristics derived from excision specimens. More recently, other features and models are assessed for their role to indicate systemic therapy. For example, plans exist to add the human epidermal growth factor receptor 2 (HER2/neu) marker in the AOL model, and recent developments have given reason to believe that the antigen Ki-67 enhances confidence in prediction of the indication for systemic therapy in breast tumors [33]. A more recent model that considers HER2/neu, Ki-67 and the mode of detection is PREDICT [34]. In our study, HER2/neu, Ki-67 and the mode of detection were not available. Future studies may provide evidence whether models like PREDICT could raise agreement in preoperative prognosis. On the other hand, agreement of HER2/neu status between LCNB and the excision specimen is reported to be as low as 60% [20]. Conversely, it may be beneficial to add a pretreatment feature as an adjunct to prediction models like AOL.

Possibly, *in vivo* functional imaging characteristics of the tumor could be employed to improve the agreement on indication for systemic therapy. MR imaging, for example, provides an overview of the entire tumor *in vivo* with potential to visualize its biological behavior, provide functional tumor characteristics, as well as accurate information on tumor extent [21]–[23], [35], [36].

## Conclusions

Using biopsied tissue and preoperative imaging as surrogates for the surgical excision specimen, a positive indication for systemic therapy based on preoperative use of AOL is likely to correspond with a positive indication postoperatively. This agreement is, however, relatively poor when preoperative AOL suggests a negative indication. In this subgroup (63% of patients), additional stratification by preoperative SNB raises the agreement. Nevertheless, preoperative assessment may lead to discordance in approximately 1 out of 10 patients compared with conventional assessment of excision specimens. Agreement in indication was especially high in the presence of ER-negative status, tumor grade 3, tumor size >2,0 cm, lymph node positivity at core biopsy and a negative preoperative SNB.
